# *Plasmodium knowlesi *malaria in Vietnam: some clarifications

**DOI:** 10.1186/1475-2875-9-20

**Published:** 2010-01-18

**Authors:** Peter Van den Eede, Indra Vythilingam, Thang Ngo Duc, Hong Nguyen Van, Le Xuan Hung, Umberto D'Alessandro, Annette Erhart

**Affiliations:** 1 Department of Parasitology, Institute of Tropical Medicine, Antwerp, Belgium; 2 Institute for Medical Research, Kuala Lumpur, Malaysia; 3 National Institute of Malariology, Parasitology & Entomology, Hanoi, Vietnam

## Abstract

A recently published comment on a report of *Plasmodium knowlesi *infections in Vietnam states that this may not accurately represent the situation in the study area because the PCR primers used may cross-hybridize with *Plasmodium vivax*. Nevertheless, *P. knowlesi *infections have been confirmed by sequencing. In addition, a neighbour-joining tree based on the 18S S-Type SSUrRNA gene shows that the Vietnamese samples clearly cluster with the *P. knowlesi *isolates identified in Malaysia and are distinct from the corresponding *P. vivax *sequences. All samples came from asymptomatic individuals who did not consult for fever during the months preceding or following the survey, indicating that asymptomatic *P. knowlesi *infections occur in this population, although this does not exclude the occurrence of symptomatic cases. Large-scale studies to determine the extent and the epidemiology of *P. knowlesi *malaria in Vietnam are further needed.

## Introduction

Cox-Singh has recently published a comment on a paper recently published in the Malaria Journal [1], in which the presence of *Plasmodium knowlesi *infections in a Ra-glai community living in a forested area of central Vietnam is reported. In her comments, Cox-Singh states that this report may not accurately represent the *P. knowlesi *situation in the study area [2]. Such argument is based on the possibility of cross-hybridization of the primers used with *Plasmodium vivax*.

## Discussion

Cross-hybridization of the primers used with *P. vivax *is a well known problem and Cox-Singh fails to mention that all *P. knowlesi *samples detected by PCR were cloned and sequenced in order to confirm the presence of this parasite [1]. Indeed, as already reported, several individuals initially positive by PCR for *P. knowlesi *were subsequently not confirmed by sequencing due to unspecific reactions with human DNA [1], indicating an inherent limitation of the PCR technique originally published by the Sarawack group [3]. This is further confirmed by the cross-hybridization with *P. vivax *recently published by Imwong *et al *[4].

**Figure 1:**
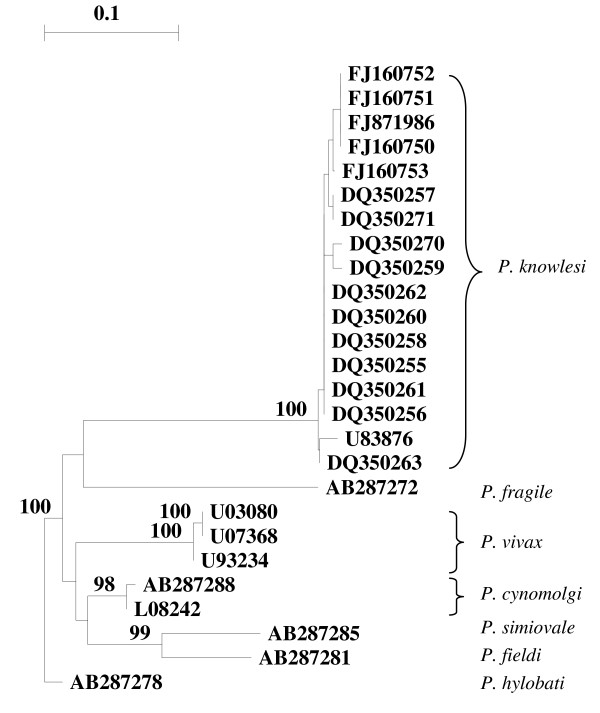
**Neighbour-joining tree based on the 18S SSUrRNA gene sequences of *P. knowlesi*, *P. vivax *and other plasmodia**. The tree was constructed with Treecon [7]. Bootstrap percentages based on 1,000 replicates are shown on the branches (if > 75%).

Both the accession numbers of sequences used by Van den Eede *et al *and their similarity with the corresponding *P. knowlesi *18S S-Type SSUrRNA gene sequences available on genbank were provided [1]. Additional evidence against the cross-hybridization hypothesized by Cox-Singh is provided by the neighbour-joining tree based on the 18S S-Type SSUrRNA gene showing that the five Vietnamese samples (FJ160750, FJ160751, FJ160752, FJ160753 and FJ871986) clearly cluster with the *P. knowlesi *isolates identified in Malaysia and are distinct from the corresponding *P. vivax *sequences (Figure 1).

Cox-Singh also stated that Van den Eede *et al *[1] failed to discover symptomatic *P. knowlesi *cases in the human population. This is a misinterpretation as the objective of the study had never been to look for symptomatic *P. knowlesi *cases. Indeed, as explained in the initial section, screening for *P. knowlesi *infections was carried out after finding an unexpectedly high number of *Plasmodium malariae *infections (mainly as mixed infections), when analysing blood samples collected during the 2004 cross-sectional survey using species-specific PCR [1]. In addition, the local conditions (environment, vector, host and human behaviour) were similar to those previously described by Cox-Singh for the natural transmission of *P. knowlesi *[5]. Therefore, the aim of the study carried out in Vietnam was not to describe the prevalence or the epidemiology of *P. knowlesi*, but rather confirm its presence in the study area. Contrary to what has been stated by Cox-Singh, the conclusion by Van den Eede *et al *was not of a low *P. knowlesi *prevalence rather that probably this infection is relatively common given the finding of one child being still infected at one year interval.

*Anopheles dirus s.s*., a member of the *Anopheles leucospherus *group, is the main malaria vector in Central Vietnam, and one specimen infected with *P. knowlesi *has been recently reported from the neighbouring province of Khan Hoa [6]. This is not surprising since the southern half of Vietnam belongs to the geographical range of the long-tailed and pig-tailed macaques, the natural hosts of *P. knowlesi *[5]. Macaques are present in the area where the study reported by Van den Eede *et al *has been carried out and are in close contact with humans who often keep them as pets. *Plasmodium knowlesi*-infected monkeys have not been reported yet in Vietnam, but this does not exclude the possibility of a natural transmission cycle. At the time when the first large human focus of *P. knowlesi *infections in Malaysian Borneo was published in 2004, neither the vector nor the natural host infected with *P. knowlesi *had been found [3].

Samples collected in Vietnam on symptomatic cases were not genotyped. Nevertheless, samples collected during the two cross-sectional surveys and identified as having a *P. knowlesi *infection came from individuals who were asymptomatic at the time of the survey and did not consult for fever during the months preceding or following the survey. This is an indication that asymptomatic *P. knowlesi *infections occur in this population, without excluding the presence of symptomatic cases. This also supports the hypothesis of a *P. knowlesi *human reservoir of infections raised by Singh *et al *in 2004 [3].

## Conclusions

The three human *P. knowlesi *infections reported from central Vietnam are just "case reports", without any pretence of describing the epidemiology of this simian species. Nevertheless, these findings, i.e. *P. knowlesi *occurring in young children and as asymptomatic infection, contribute to the body of knowledge on *P. knowlesi *epidemiology in South East Asia and cannot be easily dismissed as non-representative. Large-scale studies to determine the extent and the epidemiology of *P. knowlesi *malaria in Vietnam are further needed.

## Conflict of interests

The authors declare that they have no competing interests.

## Authors' contributions

PVDE carried out the PCR analysis and data analysis; HNV helped in the PCR analysis. IV kindly provided the training and expertise for the set up of the *P. knowlesi *at the Department of Parasitology, ITM Antwerp and made substantial to reviewing the paper. AE and UDA made substantial contributions to conceive the study design, paper writing and reviewing. NDT, LXH, and AE coordinated the cluster randomized trial in Ninh Thuan province, and revised the manuscript. All authors read and approved the final manuscript.
